# On Ev-Degree and Ve-Degree Topological Properties of Tickysim Spiking Neural Network

**DOI:** 10.1155/2019/8429120

**Published:** 2019-06-02

**Authors:** Murat Cancan

**Affiliations:** Faculty of Education, Van Yüzüncü Yıl University, 65080 Van, Turkey

## Abstract

Topological indices are indispensable tools for analyzing networks to understand the underlying topology of these networks. Spiking neural network architecture (SpiNNaker or TSNN) is a million-core calculating engine which aims at simulating the behavior of aggregates of up to a billion neurons in real time. Tickysim is a timing-based simulator of the interchip interconnection network of the SpiNNaker architecture. Tickysim spiking neural network is considered to be highly symmetrical network classes. Classical degree-based topological properties of Tickysim spiking neural network have been recently determined. Ev-degree and ve-degree concepts are two novel degrees recently defined in graph theory. Ev-degree and ve-degree topological indices have been defined as parallel to their corresponding counterparts. In this study, we investigate the ev-degree and ve-degree topological properties of Tickysim spiking neural network. These calculations give the information about the underlying topology of Tickysim spiking neural network.

## 1. Introduction

Biological neurons use signals that are known as action potentials, membrane potentials, or spikes in short and sudden increments to transmit information to neighboring neurons. Spiking neural network (SNN), a special class of artificial neural networks, communicates with the spikes of the neuron models. Spiking neural network systems are a class of parallel neural-like computing and distributed models which are inspired from the way neurons communicate by means of spikes. Significant amounts of data can be processed by networks of spiking neurons, using relatively few spikes [1]. Spiking models that provide powerful tools for the analysis of basic operations, including neural information processing, plasticity, and learning in the brain, are very similar to biological neurons. In practical engineering, solutions for a wide variety of specific problems, such as fast signal processing, event detection, classification, speech recognition, spatial navigation, or motor control, are provided by spiking networks [2]. SNN has also been shown to be more computationally stronger than sensors and sigmoidal gates [3]. SNNs in neuromorphic hardware show positive characteristics such as low power consumption, rapid extraction, and event-based information processing. This makes them interesting candidates for the efficient application of deep neural networks, the preferred method for many machine learning tasks [4].

Spiking neural network architecture (SpiNNaker or TSNN) is a million-core calculating engine which aims at simulating the behavior of aggregates of up to a billion neurons in real time. Tickysim is a timing-based simulator of the interchip interconnection network of the SpiNNaker (TSNN) architecture. Tickysim spiking neural network is considered to be highly symmetrical network classes. For an overview of the SpiNNaker system architecture, we refer the interested reader to reference [5]. Quaternary synapses network for memristor-based spiking convolutional neural networks has been investigated in [6]. Spiking neural *p* systems with learning functions have been proposed in [7]. Spiking neural networks for handwritten digit recognition-supervised learning and network optimization has been investigated in [8]. Generalized matrix inverse on spiking neural substrate has been calculated in [9].

Neuromorphic computing is very important in view of the data processing, machine learning, and artificial intelligence. This computing gives information about the underlying topology of neural networks. Among these computations, the computation of classical degree-based topological properties of the Tickysim spiking neural network has newly been investigated in [10]. In this study, we investigate the ev-degree and ve-degree topological properties of the Tickysim spiking neural network as a continuation of the study [10].

**Figure 1:**
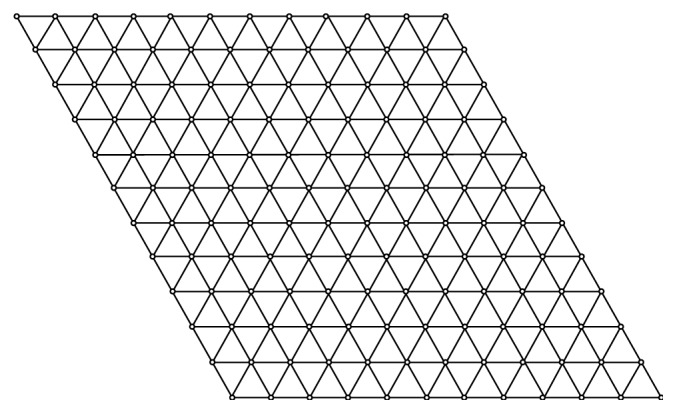
Graph of the Tickysim spiking neural network for *m*=*n*=12.

The graph of a Tickysim spiking neural network sheet is shown in Figure 1. The number of vertices in the Tickysim spiking neural network is represented as *m* × *n*.

Ev-degree and ve-degree concepts are two novel degrees recently defined in graph theory [11]. Ev-degree and ve-degree topological indices have been defined as parallel to their corresponding counterparts [12–15].

## 2. Preliminaries

In this section, we give some basic and preliminary concepts which we shall use later. A graph *G = (V, E)* consists of two nonempty sets *V* and 2-element subsets of *V*, namely, *E*. The elements of *V* are called vertices, and the elements of *E* are called edges. For a vertex *v*, deg(*v*) shows the number of edges that is incident to *v*. The set of all vertices which is adjacent to *v* is called the open neighborhood of *v* and denoted by *N(v)*. If we add the vertex *v* to *N(v)*, then we get the closed neighborhood of *v*, *N[v]*. And now, we give the definitions of ev-degree and ve-degree concepts which were given in [11].

**Definition 1 (ve-degree).**

Let *G* be a connected simple graph and *v* *∈* *V(G)*. The ve-degree of the vertex *v*, deg_ve_(*v*), equals the number of different edges that is incident to any vertex from the closed neighborhood of *v*.

We also can restate Definition 1 as follows: Let *G* be a connected simple graph and *v* *∈* *V(G)*. The ve-degree of the vertex *v* is the number of different edges between the other vertices with a maximum distance of two from the vertex *v*.

**Definition 2 (ev-degree).**

Let *G* be a connected graph and *e* *=* *uv* *∈* *E(G)*. The ev-degree of the edge *e*, deg_ev_(*e*), equals the number of vertices of the union of the closed neighborhoods of *u* and *v*.

The authors in [11] also can give the Definition 2 as follows: Let *G* be a connected graph and *e* *=* *uv* *∈* *E(G).* The ev-degree of the edge *e*, deg_uv_(*e*) *=* deg*u* *+* deg*v*−*n*_*e*_, where *n*_*e*_ means the number of triangles in which the edge *e* lies in.

**Definition 3 (ev-degree Zagreb index).**

Let *G* be a connected graph and *e* *=* *uv* *∈* *E(G).* The ev-degree Zagreb index of the graph *G* defined as

(1) $$\begin{array}{c} M^{\text{ev}}\left(G\right)=\underset{e\in E\left(G\right)}{\sum }{\text{deg}}_{\text{ev}}e^{2}. \end{array}$$

**Definition 4 (the first ve-degree Zagreb alpha index).**

Let *G* be a connected graph and *v* *∈* *V(G).* The first ve-degree Zagreb alpha index of the graph *G* defined as

(2) $$\begin{array}{c} M_{1}^{\alpha \text{ve}}\left(G\right)=\underset{v\in V\left(G\right)}{\sum }{\text{deg}}_{\text{ve}}v^{2}. \end{array}$$

**Definition 5 (the first ve-degree Zagreb beta index).**

Let *G* be a connected graph and *uv* *∈* *E(G).* The first ve-degree Zagreb beta index of the graph *G* defined as

(3) $$\begin{array}{c} M_{1}^{\beta \text{ve}}\left(G\right)=\underset{uv\in E\left(G\right)}{\sum }\left({\text{deg}}_{\text{ve}}u+{\text{deg}}_{\text{ve}}v\right). \end{array}$$

**Definition 6 (the second ve-degree Zagreb index).**

Let *G* be a connected graph and *uv* *∈* *E(G).* The second ve-degree Zagreb index of the graph *G* defined as

(4) $$\begin{array}{c} M_{2}^{\text{ve}}\left(G\right)=\underset{uv\in E\left(G\right)}{\sum }{\text{deg}}_{\text{ve}}u{\text{deg}}_{\text{ve}}v. \end{array}$$

**Definition 7 (ve-degree Randic index).**

Let *G* be a connected graph and *uv* *∈* *E(G)*. The ve-degree Randic index of the graph *G* defined as

(5) $$\begin{array}{c} R^{\text{ve}}\left(G\right)=\underset{uv\in E\left(G\right)}{\sum }{\left({\text{deg}}_{\text{ve}}u{\text{deg}}_{\text{ve}}v\right)}^{-1/2}. \end{array}$$

**Definition 8 (ev-degree Randic index).**

Let *G* be a connected graph and *e* *=* *uv* *∈* *E(G)*. The ev-degree Randic index of the graph *G* defined as

(6) $$\begin{array}{c} R^{\text{ev}}\left(G\right)=\underset{e\in E\left(G\right)}{\sum }{\text{deg}}_{\text{ev}}e^{-1/2}. \end{array}$$

**Definition 9 (ve-degree atom-bond connectivity index).**

The ve-degree atom-bond connectivity (ve-ABC) index for a connected graph *G* defined as

(7) $$\begin{array}{c} {\text{ABC}}^{\text{ve}}\left(G\right)=\underset{uv\in E\left(G\right)}{\sum }\sqrt{\frac{{\text{deg}}_{\text{ve}}\left(u\right)+{\text{deg}}_{\text{ve}}\left(v\right)-2}{{\text{deg}}_{\text{ve}}\left(u\right)\times {\text{deg}}_{\text{ve}}\left(v\right)}}. \end{array}$$

**Definition 10 (ve-degree geometric-arithmetic index).**

The ve-degree geometric-arithmetic (ve-GA index) for a connected graph *G* defined as

(8) $$\begin{array}{c} {\text{GA}}^{\text{ve}}\left(G\right)=\underset{uv\in E\left(G\right)}{\sum }\frac{2\sqrt{{\text{deg}}_{\text{ve}}\left(u\right)\times {\text{deg}}_{\text{ve}}\left(v\right)}}{{\text{deg}}_{\text{ve}}\left(u\right)+{\text{deg}}_{\text{ve}}\left(v\right)}. \end{array}$$

**Definition 11 (ve-degree harmonic index).**

The ve-degree harmonic (ve-*H*) index for a connected graph *G* defined as

(9) $$\begin{array}{c} H^{\text{ve}}\left(G\right)=\underset{uv\in E\left(G\right)}{\sum }\frac{2}{{\text{deg}}_{\text{ve}}\left(u\right)+{\text{deg}}_{\text{ve}}\left(v\right)}. \end{array}$$

**Definition 12 (ve-degree sum-connectivity index).**

The ve-degree sum-connectivity (ve-*χ*) index for a connected graph *G* defined as

(10) $$\begin{array}{c} \chi^{\text{ve}}\left(G\right)=\underset{uv\in E\left(G\right)}{\sum }{\left({\text{deg}}_{\text{ve}}\left(u\right)+{\text{deg}}_{\text{ve}}\left(v\right)\right)}^{-1/2}. \end{array}$$

## 3. Results

We know that the Tickysim spiking neural network has *m*  ×  *n* vertices and 3*mn* − 2*m* − 2*n*+1 edges. With the help of Figure 1 and Table 1, we give the ev-degree partition of the edges of the Tickysim spiking neural network.

And from Tables 2 and 3, we give the ve-degree partition of the vertices of the Tickysim spiking neural network.

The ev-degree and ve-degree topological indices of the Tickysim spiking neural network are given in Table 4.

## 4. Conclusions

Neuromorphic computing is very important in view of the data processing, machine learning, and artificial intelligence. This computing gives information about the underlying topology of neural networks. Among these computations, the computation of the ev-degree and ve-degree topological properties of Tickysim spiking neural network provided the information about the underlying topology of the Tickysim spiking neural network. The other well-known topological indices values of the Tickysim spiking neural network are interesting for further studies. Also, the mathematical properties of ev-degree and ve-degree topological indices have not been studied so far. In this regard, the mathematical properties are worth to investigate for future studies.

## Figures and Tables

**Table 1:** The ev-degree of the edges of the Tickysim spiking neural network.

Number of edges	Degrees of its end vertices	Ev-degrees
4	(2,4)	5
4	(3,4)	6
2	(3,6)	7
2	(4,4)	6
2*m*+2*n* − 12	(4,4)	7
4*m*+4*n* − 20	(4,6)	8
3*mn* − 8*m* − 8*n*+21	(6,6)	10

**Table 2:** The ve-degree of the vertices of the Tickysim spiking neural network.

Number of vertices	Degrees	Ve-degrees
2	2	9
2	3	16
4	4	16
4	4	21
2*m*+2*n* − 16	4	23
2	6	26
2	6	29
8	6	34
2*m* − 12	6	35
2*n* − 12	6	36
*mn* − 4*m* − 4*n*+16	6	42

**Table 3:** The ve-degree of the end vertex of edges of the Tickysim spiking neural network.

Number of edges	Degrees of its end vertices	Ve-degrees of its end vertices
4	(2,4)	(9,16)
4	(3,4)	(16,21)
2	(3,6)	(16,29)
2	(4,4)	(16,16)
2	(4,4)	(16,23)
2	(4,4)	(21,23)
2*m*+2*n* − 16	(4,4)	(23,23)
4	(4,6)	(16,26)
4	(4,6)	(21,29)
4	(4,6)	(23,26)
2	(4,6)	(21,34)
4	(4,6)	(23,34)
2*m*	(4,6)	(23,35)
2*m*+4*n* − 38	(4,6)	(23,36)
4	(6,6)	(26,34)
4	(6,6)	(29,34)
2	(6,6)	(34,34)
2	(6,6)	(29,42)
12	(6,6)	(34,42)
4	(6,6)	(34,35)
2*m* − 12	(6,6)	(35,35)
4	(6,6)	(34,36)
2*n* − 14	(6,6)	(36,36)
*m*	(6,6)	(35,42)
*n*	(6,6)	(36,42)
3*mn* − 11*m* − 11*n*+15	(6,6)	(42,42)

And we begin to compute ev-degree and ve-degree topological indices.

**Table 4:** The topological indices of the Tickysim spiking neural network.

Topological index	Topological index value of the Tickysim spiking neural network (TSNN)
Ev-degree Zagreb index *M*^ev^(TSSN)	300*mn* − 446*m* − 446*n*+354
The first ve-degree Zagreb alpha index *M*_1_^*α*ve^(TSNN)	1764*mn* − 3548*m* − 3406*n* − 5252
The first ve-degree Zagreb beta index *M*_1_^*β*ve^(TSSN)	252*mn* − 381*m* − 374*n*+142
The second ve-degree Zagreb index *M*_2_^ve^(TSSN)	5292*mn* − 11160*m* − 10930*n*+8760
Ve-degree Randic index *R*^ve^(TSSN)	$\begin{array}{c} \left(1/3\right)+\left(1/\sqrt{21}\right)+\left(1/2\sqrt{29}\right)+\left(1/8\right)+\left(1/2\sqrt{23}\right)+\left(2/\sqrt{483}\right)+\left(\left(2m+2n-16\right)/23\right)+1/\sqrt{26}+\left(4/\sqrt{609}\right)+\left(4/\sqrt{598}\right)+\left(2/\sqrt{714}\right)+\left(4/\sqrt{782}\right)+\left(2m/\sqrt{805}\right)+\\ \left(\left(2m+4n-38\right)/6\sqrt{23}\right)+\left(2/\sqrt{221}\right)+\left(4/\sqrt{986}\right)+\left(1/17\right)+\left(2/\sqrt{1218}\right)+\left(6/\sqrt{357}\right)+\left(4/\sqrt{1190}\right)+\left(\left(2m-12\right)/35\right)+\left(2/3\sqrt{34}\right)+\left(\left(n-7\right)/18\right)+\left(m/7\sqrt{30}\right)+\left(n/6\sqrt{42}\right)+\left(\left(3mn-11m-11n+15\right)/42\right) \end{array}$
Ev-degree Randic index *R*^ev^(TSSN)	$\left(4/\sqrt{5}\right)+\left(6/\sqrt{6}\right)+\left(\left(2m+2n-10\right)/\sqrt{7}\right)+\left(\left(4m+4n-20\right)/\sqrt{8}\right)+\left(\left(43mn-8m-8n+21\right)/\sqrt{10}\right)$
Ve-degree atom-bond connectivity index ABC^ve^(TSSN)	$\begin{array}{c} \left(\sqrt{23}/3\right)+\left(\sqrt{35}/\sqrt{21}\right)+\left(\sqrt{43}/2\sqrt{29}\right)+\left(\sqrt{30}/8\right)+\left(\sqrt{37}/2\sqrt{23}\right)+\left(2\sqrt{42}/\sqrt{483}\right)+\left(\left(2\left(2m+2n-16\right)\sqrt{11}\right)/23\right)+\left(2\sqrt{10}/\sqrt{26}\right)+\left(16\sqrt{3}/\sqrt{609}\right)+\\ \left(4\sqrt{47}/\sqrt{598}\right)+\left(2\sqrt{53}/\sqrt{714}\right)+\left(4\sqrt{55}/\sqrt{782}\right)+\left(2m\sqrt{56}/\sqrt{805}\right)+\left(\left(\left(2m+4n-38\right)\sqrt{57}\right)/6\sqrt{23}\right)+\left(2\sqrt{58}/\sqrt{221}\right)+\\ \left(4\sqrt{61}/\sqrt{986}\right)+\left(\sqrt{66}/17\right)+\left(2\sqrt{69}/\sqrt{1218}\right)+\left(6\sqrt{74}/\sqrt{357}\right)+\left(4\sqrt{67}/\sqrt{1190}\right)+\left(\left(2\left(2m-12\right)\sqrt{17}\right)/35\right)+\left(4\sqrt{17}/3\sqrt{34}\right)+\left(\left(n-7\right)\sqrt{70}/18\right)+\left(\left(5m\sqrt{3}\right)/7\sqrt{30}\right)+\left(\left(2\sqrt{19}n\right)/6\sqrt{42}\right)+\left(\left(\left(3mn-11m-11n+15\right)\sqrt{82}\right)/42\right) \end{array}$
Ve-degree geometric-arithmetic index GA^ve^(TSSN)	$\begin{array}{c} 3mn-7m-7n+\left(\left(2m\sqrt{805}\right)/29\right)+\left(\left(2m\sqrt{30}\right)/11\right)+\left(\left(6n\sqrt{42}\right)/39\right)+\left(\left(\left(24m+48n-456\right)\sqrt{23}\right)/59\right)-23+\left(96/25\right)+\left(64\sqrt{21}/37\right)\\ +\left(16\sqrt{29}/45\right)+\left(16\sqrt{23}/39\right)+\left(\sqrt{483}/11\right)+\left(16\sqrt{26}/21\right)+\left(4\sqrt{609}/25\right)+\left(8\sqrt{598}/49\right)+\left(4\sqrt{714}/55\right)+\left(8\sqrt{782}/57\right)\\ +\left(4\sqrt{221}/15\right)+\left(8\sqrt{986}/63\right)+\left(4\sqrt{1218}/71\right)+\left(12\sqrt{357}/19\right)+\left(8\sqrt{1190}/69\right)+\left(24\sqrt{34}/35\right) \end{array}$
Ve-degree harmonic index *H*^ve^(TSSN)	$\begin{array}{c} \left(8/25\right)+\left(8/37\right)+\left(4/45\right)+\left(1/8\right)+\left(4/39\right)+\left(1/11\right)+\left(\left(2m+2n-16\right)/23\right)+\left(4/21\right)+\left(4/25\right)+\left(8/49\right)+\left(4/55\right)\\ +\left(8/57\right)+\left(2m/29\right)+\left(\left(\left(2m+4n-38\right)x2\right)/59\right)+\left(2/15\right)+\left(8/63\right)+\left(1/17\right)+\left(4/71\right)+\left(6/19\right)+\left(8/69\right)+\left(\left(2m-12\right)/35\right)+\left(4/35\right)+\left(\left(n-7\right)/18\right)+\left(2m/77\right)+\left(n/39\right)+\left(\left(3mn-11m-11n+15\right)/42\right) \end{array}$
Ve-degree sum-connectivity index *χ*^ve^(TSSN)	$\begin{array}{c} \left(4/5\right)+\left(4/\sqrt{37}\right)+\left(2/3\sqrt{5}\right)+\left(1/2\sqrt{2}\right)+\left(2/\sqrt{39}\right)+\left(1/\sqrt{11}\right)+\left(\left(2m+2n-16\right)/\sqrt{46}\right)+\left(4/\sqrt{42}\right)\\ +\left(4/5\sqrt{2}\right)+\left(4/7\right)+\left(2/\sqrt{55}\right)+\left(4/\sqrt{57}\right)+\left(2m/\sqrt{58}\right)+\left(\left(2m+4n-38\right)/\sqrt{59}\right)+\left(2/\sqrt{15}\right)+\left(4/3\sqrt{7}\right)\\ +\left(1/\sqrt{17}\right)+\left(2/\sqrt{71}\right)+\left(6/\sqrt{19}\right)+\left(4/\sqrt{69}\right)+\left(\left(2m-12\right)/\sqrt{70}\right)+\left(4/\sqrt{70}\right)+\left(\left(2n-14\right)/6\sqrt{2}\right)+\left(m/\sqrt{77}\right)+\left(n/\sqrt{78}\right)+\left(\left(3mn-11m-11n+15\right)/2\sqrt{21}\right) \end{array}$

## Data Availability

The data used to support the findings of this study are included within the article.

## Appendix

### The Calculations in Table 4

The following calculations (A.1)–(A.10) were made by using Figure 1, Tables 1, 2, and 3, and the definitions of ev-degree and ve-degree topological indices:

(A.1) $$\begin{array}{c} M^{\text{ev}}\left(\text{TSSN}\right)=\underset{e\in E\left(\text{TSSN}\right)}{\sum }\deg_{\text{ev}}e^{2}=4x5^{2}+4x6^{2}+2x7^{2}+2x6^{2}+\left(2m+2n-12\right)x7^{2}+\left(4m+4n-20\right)x8^{2}+\left(3mn-8m-8n+21\right)x10^{2}=300mn-446m-446n+354, \end{array}$$

(A.2) $$\begin{array}{c} M_{1}^{\alpha \text{ve}}\left(\text{TSSN}\right)=\underset{v\in V\left(\text{TSSN}\right)}{\sum }\deg_{\text{ve}}v^{2}=2x9^{2}+2x16^{2}+4x16^{2}+4x21^{2}+\left(2m+2n-16\right)x23^{2}+2x26^{2}+2x29^{2}+8x34^{2}+\left(2m-12\right)x35^{2}+\left(2n-12\right)x36^{2}+\left(mn-4m-4n+16\right)x42^{2}=1764mn-3548m-3406n-5252, \end{array}$$

(A.3) $$\begin{array}{c} M_{1}^{\beta }\left(\text{TSSN}\right)=\underset{uv\in E\left(\text{TSSN}\right)}{\sum }\left(\deg_{\text{ve}}u+\deg_{\text{ve}}v\right)=4x25+4x37+2x45+2x32+2x39+2x44+\left(2m+2n-16\right)x46+4x42+4x50+4x49+2x55+4x57+2mx58+\left(2m+4n-38\right)x59+4x60+4x63+2x68+2x71+12x76+4x69+\left(2m-12\right)x70+4x70+\left(2n-14\right)x72+mx77+nx78+\left(3mn-11m-11n+15\right)x84=252mn-381m-374n+142, \end{array}$$

(A.4) $$\begin{array}{c} M_{2}^{\text{ve}}\left(TSSN\right)=\underset{uv\in E\left(TSSN\right)}{\sum }\deg_{\text{ve}}u\deg_{\text{ve}}v=4x9x16+4x16x21+2x16x29+2x16x16+2x16x23+2x21x23+\left(2m+2n-16\right)x23x23+4x16x26+4x21x29+4x23x26+2x21x34+4x23x34+2mx23x35+\left(2m+4n-38\right)x23x36+4x26x34+4x29x34+2x34x34+2x29x42+12x34x42+4x34x35+\left(2m-12\right)x35x35+4x34x36+\left(2n-14\right)x36x36+mx35x42+nx36x42+\left(3mn-11m-11n+15\right)x42x42=5292mn-11160m-10930n+8760, \end{array}$$

(A.5) $$\begin{array}{c} R^{\text{ve}}\left(\text{TSSN}\right)=\underset{uv\in E\left(\text{TSSN}\right)}{\sum }{\left(\deg_{\text{ve}}u\deg_{\text{ve}}v\right)}^{-1/2}=\frac{4}{\sqrt{9x16}}+\frac{4}{\sqrt{16x21}}+\frac{2}{\sqrt{16x29}}+\frac{2}{\sqrt{16x16}}+\frac{2}{\sqrt{23x16}}+\frac{2}{\sqrt{21x23}}+\frac{2m+2n-16}{\sqrt{23x23}}+\frac{4}{\sqrt{16x26}}+\frac{4}{\sqrt{21x29}}+\frac{4}{\sqrt{23x26}}+\frac{2}{\sqrt{21x34}}+\frac{4}{\sqrt{23x34}}+\frac{2m}{\sqrt{23x35}}+\frac{2m+4n-38}{\sqrt{23x36}}+\frac{4}{\sqrt{26x34}}+\frac{4}{\sqrt{29x34}}+\frac{2}{\sqrt{34x34}}+\frac{2}{\sqrt{29x42}}+\frac{12}{\sqrt{34x42}}+\frac{4}{\sqrt{34x35}}+\frac{2m-12}{\sqrt{35x35}}+\frac{4}{\sqrt{34x36}}+\frac{2n-14}{\sqrt{36x36}}+\frac{m}{\sqrt{35x42}}+\frac{n}{\sqrt{36x42}}+\frac{3mn-11m-11n+15}{\sqrt{42x42}}, \end{array}$$

(A.6) $$\begin{array}{c} R^{\text{ev}}\left(\text{TSSN}\right)=\underset{e\in E\left(\text{TSSN}\right)}{\sum }\deg_{\text{ev}}e^{-1/2}=\frac{4}{\sqrt{5}}+\frac{4}{\sqrt{6}}+\frac{2}{\sqrt{7}}+\frac{2}{\sqrt{6}}+\frac{2m+2n-12}{\sqrt{7}}+\frac{4m+4n-20}{\sqrt{8}}+\frac{43mn-8m-8n+21}{\sqrt{10}}=\frac{4}{\sqrt{5}}+\frac{6}{\sqrt{6}}+\frac{2m+2n-10}{\sqrt{7}}+\frac{4m+4n-20}{\sqrt{8}}+\frac{43mn-8m-8n+21}{\sqrt{10}}, \end{array}$$

(A.7) $$\begin{array}{c} {\text{ABC}}^{\text{ve}}\left(\text{TSSN}\right)=\underset{uv\in E\left(\text{TSSN}\right)}{\sum }\sqrt{\frac{\deg_{\text{ve}}\left(u\right)+\deg_{\text{ve}}\left(v\right)-2}{\deg_{\text{ve}}\left(u\right)\times \deg_{\text{ve}}\left(v\right)}}=\frac{4\sqrt{23}}{\sqrt{9x16}}+\frac{4\sqrt{35}}{\sqrt{16x21}}+\frac{2\sqrt{43}}{\sqrt{16x29}}+\frac{2\sqrt{30}}{\sqrt{16x16}}+\frac{2\sqrt{37}}{\sqrt{23x16}}+\frac{2\sqrt{42}}{\sqrt{21x23}}+\frac{\left(2m+2n-16\right)\sqrt{44}}{\sqrt{23x23}}+\frac{4\sqrt{40}}{\sqrt{16x26}}+\frac{4\sqrt{48}}{\sqrt{21x29}}+\frac{4\sqrt{47}}{\sqrt{23x26}}+\frac{2\sqrt{53}}{\sqrt{21x34}}+\frac{4\sqrt{55}}{\sqrt{23x34}}+\frac{2m\sqrt{56}}{\sqrt{23x35}}+\frac{\left(2m+4n-38\right)\sqrt{57}}{\sqrt{23x36}}+\frac{4\sqrt{58}}{\sqrt{26x34}}+\frac{4\sqrt{61}}{\sqrt{29x34}}+\frac{2\sqrt{66}}{\sqrt{34x34}}+\frac{2\sqrt{69}}{\sqrt{29x42}}+\frac{12\sqrt{74}}{\sqrt{34x42}}+\frac{4\sqrt{67}}{\sqrt{34x35}}+\frac{\left(2m-12\right)\sqrt{68}}{\sqrt{35x35}}+\frac{4\sqrt{68}}{\sqrt{34x36}}+\frac{\left(2n-14\right)\sqrt{70}}{\sqrt{36x36}}+\frac{m\sqrt{75}}{\sqrt{35x42}}+\frac{n\sqrt{76}}{\sqrt{36x42}}+\frac{\left(3mn-11m-11n+15\right)\sqrt{82}}{\sqrt{42x42}} \end{array}$$

(A.8) $$\begin{array}{c} {\text{GA}}^{\text{ve}}\left(\text{TSSN}\right)=\underset{uv\in E\left(\text{TSSN}\right)}{\sum }\frac{2\sqrt{\deg_{\text{ve}}\left(u\right)\times \deg_{\text{ve}}\left(v\right)}}{\deg_{\text{ve}}\left(u\right)+\deg_{\text{ve}}\left(v\right)}=\frac{4x2\sqrt{9x16}}{25}+\frac{4x4\sqrt{16x21}}{37}+\frac{2x2\sqrt{16x29}}{45}+\frac{2x2\sqrt{16x16}}{32}+\frac{2x2\sqrt{23x16}}{39}+\frac{2x2\sqrt{21x23}}{44}+\frac{2x\left(2m+2n-16\right)\sqrt{23x23}}{46}+\frac{4x2\sqrt{16x26}}{42}+\frac{4x2\sqrt{21x29}}{50}+\frac{4x2\sqrt{23x26}}{49}+\frac{2x2\sqrt{21x34}}{55}+\frac{4x2\sqrt{23x34}}{57}+\frac{2mx2\sqrt{23x35}}{58}+\frac{\left(2m+4n-38\right)x2\sqrt{23x36}}{59}+\frac{4x2\sqrt{26x34}}{60}+\frac{4x2\sqrt{29x34}}{63}+\frac{2x2\sqrt{34x34}}{68}+\frac{2x2\sqrt{29x42}}{71}+\frac{12x2\sqrt{34x42}}{76}+\frac{4x2\sqrt{34x35}}{69}+\frac{\left(2m-12\right)x2\sqrt{35x35}}{70}+\frac{4x2\sqrt{34x36}}{70}+\frac{\left(2n-14\right)x2\sqrt{36x36}}{72}+\frac{mx2\sqrt{35x42}}{77}+\frac{nx2\sqrt{36x42}}{78}+\frac{\left(3mn-11m-11n+15\right)x2\sqrt{42x42}}{84} \end{array}$$

(A.9) $$\begin{array}{c} H^{\text{ve}}\left(\text{TSSN}\right)=\underset{uv\in E\left(S_{n}\right)}{\sum }\frac{2}{\deg_{\text{ve}}\left(u\right)+\deg_{\text{ve}}\left(v\right)}=\frac{4x2}{25}+\frac{4x2}{37}+\frac{2x2}{45}+\frac{2x2}{32}+\frac{2x2}{39}+\frac{2x2}{44}+\frac{\left(2m+2n-16\right)x2}{46}+\frac{4x2}{42}+\frac{4x2}{50}+\frac{4x2}{49}+\frac{2x2}{55}+\frac{4x2}{57}+\frac{2mx2}{58}+\frac{\left(2m+4n-38\right)x2}{59}+\frac{4x2}{60}+\frac{4x2}{63}+\frac{2x2}{68}+\frac{2x2}{71}+\frac{12x2}{76}+\frac{4x2}{69}+\frac{\left(2m-12\right)x2}{70}+\frac{4x2}{70}+\frac{\left(2n-14\right)x2}{72}+\frac{mx2}{77}+\frac{nx2}{78}+\frac{\left(3mn-11m-11n+15\right)x2}{84} \end{array}$$

(A.10) $$\begin{array}{c} \chi^{\text{ve}}\left(\text{TSSN}\right)=\underset{uv\in E\left(S_{n}\right)}{\sum }{\left(\deg_{\text{ve}}\left(u\right)+\deg_{\text{ve}}\left(v\right)\right)}^{-1/2}=\frac{4}{\sqrt{25}}+\frac{4}{\sqrt{37}}+\frac{2}{\sqrt{45}}+\frac{2}{\sqrt{32}}+\frac{2}{\sqrt{39}}+\frac{2}{\sqrt{44}}+\frac{\left(2m+2n-16\right)}{\sqrt{46}}+\frac{4}{\sqrt{42}}+\frac{4}{\sqrt{50}}+\frac{4}{\sqrt{49}}+\frac{2}{\sqrt{55}}+\frac{4}{\sqrt{57}}+\frac{2m}{\sqrt{58}}+\frac{\left(2m+4n-38\right)}{\sqrt{59}}+\frac{4}{\sqrt{60}}+\frac{4}{\sqrt{63}}+\frac{2}{\sqrt{68}}+\frac{2}{\sqrt{71}}+\frac{12}{\sqrt{76}}+\frac{4}{\sqrt{69}}+\frac{\left(2m-12\right)}{\sqrt{70}}+\frac{4}{\sqrt{70}}+\frac{\left(2n-14\right)}{\sqrt{72}}+\frac{m}{\sqrt{77}}+\frac{n}{\sqrt{78}}+\frac{\left(3mn-11m-11n+15\right)}{\sqrt{84}} \end{array}$$
